# Broadband photoresponse data of transparent all-oxide photovoltaics of ZnO/NiO

**DOI:** 10.1016/j.dib.2019.104095

**Published:** 2019-06-12

**Authors:** Malkeshkumar Patel, Dong-Kyun Ban, Abhijit Ray, Joondong Kim

**Affiliations:** a Department of Electrical Engineering, Incheon National University, 119 Academy Rd. Yeonsu, Incheon, 22012, Republic of Korea; b Photoelectric and Energy Device Application Lab (PEDAL), Multidisciplinary Core Institute for Future Energies (MCIFE), Incheon National University, 119 Academy Rd. Yeonsu, Incheon, 22012, Republic of Korea; c Department of Solar Energy, Pandit Deendayal Petroleum University, Raisan, Gandhinagar 382007, Gujarat, India

**Keywords:** Transparent optoelectronics, All-oxide photovoltaics, Optical property, Transient photovoltage, Spectral analysis

## Abstract

In this data article, the properties of all transparent metal oxide of ZnO/NiO heterostructure “Transparent all-oxide photovoltaics and broadband high-speed energy-efficient optoelectronics” [1] are presented by characteristics of ZnO and NiO layers, open circuit voltage decay (OCVD), broadband light with intensity dependent current-voltage plots. The device performances under the effect of various optical excitation of intermediated-band, bound excitonic, free-excitonic and band-to-band are presented. The ZnO/NiO heterostructure direction grown on ITO/glass substrate by large area sputtering method [1] was characterized by UV–visible plots and scanning electron microscope (SEM). Carrier lifetime using OCVD of ZnO/NiO devices with carbon paint metal contact is presented. Prolonged open circuit voltage plots under UV light intensity are shown for stability and repeatability studies. I–V characteristics of ZnO/NiO heterostructure under the light wavelength from 623 nm to 365 nm are presented for energy efficient broadband optoelectronics.

**Table:** Specifications Table

Subject area	*Physics, Electrical Engineering*
More specific subject area	*Optoelectronics, Solar Cells, Photodetector*
Type of data	*Figures*
How data was acquired	Field emission scanning electron microscope (FESEM, JOEL, JSM_7800 F) *UV–visible diffused reflectance photo spectrometer (Simadzu, UV-2600)* *Potentiostat/galvanostat (PGStat, ZIVE SP2, WonA Tech)* *Function generator (MFG-3013A, MCH Instruments)* *Oscilloscope (*2 GHz *bandwidth, TBS 1102B-EDU, Tektronix)* *Digital photograph*
Data format	*Raw and analyzed*
Experimental factors	*Digital photograph (Ambient light condition);* *UV–visible, sample mounted on the diffused integrated sphere, scan range 800–*300 nm*;* *FESEM (samples grown on eagle glass substrate);* *OCVD (Oscilloscope high resolution auto data acquisition mode, device under* 385 nm *wavelength of light, intensity* 7 mW/cm^2^*, pulse frequency* 20 Hz*);* *Prolonged V*_*OC*_*(Oscilloscope high resolution auto data acquisition mode, device under* 385 nm *wavelength of light, intensity of 10 and* 20 mW/cm^2^*);* *Current-Voltage plots (Linear sweep voltammetry, scan range: -0.2 to 0.8V, positive direction, scan rate* 50 mV/s*, LED light wavelength of* 623 nm, 520 nm, 460 nm, 410 nm, 400 nm, 385 nm *and* 365 nm*)*
Experimental features	*Transparent, All-oxide photovoltaics NiO/ZnO device for broadband energy efficient optoelectronics*
Data source location	*Incheon National University, Incheon- 22012, South Korea*
Data accessibility	*The data are with this article. Raw data is provided as a*supplementary file*.*
Related research article	*M. Patel, D.K. Ban, A. Ray, J. Kim, Transparent all-oxide photovoltaics and broadband high-speed energy-efficient optoelectronics, Sol. Energy Mater. Sol. Cells, 194, 2019, 148–158**[1]**.*

**Table:** 

**Value of the Data.** • Photograph of the prepared ZnO/NiO devices for the transparent and large area features. • Optical data of ZnO, NiO, and ZnO/NiO device would be useful to design broadband and energy efficient transparent optoelectronic devices • Carrier lifetime, transient open circuit voltage plots and I–V characteristics of ZnO/NiO device under various light wavelength would be useful to demonstrate photovoltaic application.

## 1 Data

**Fig. 1:**
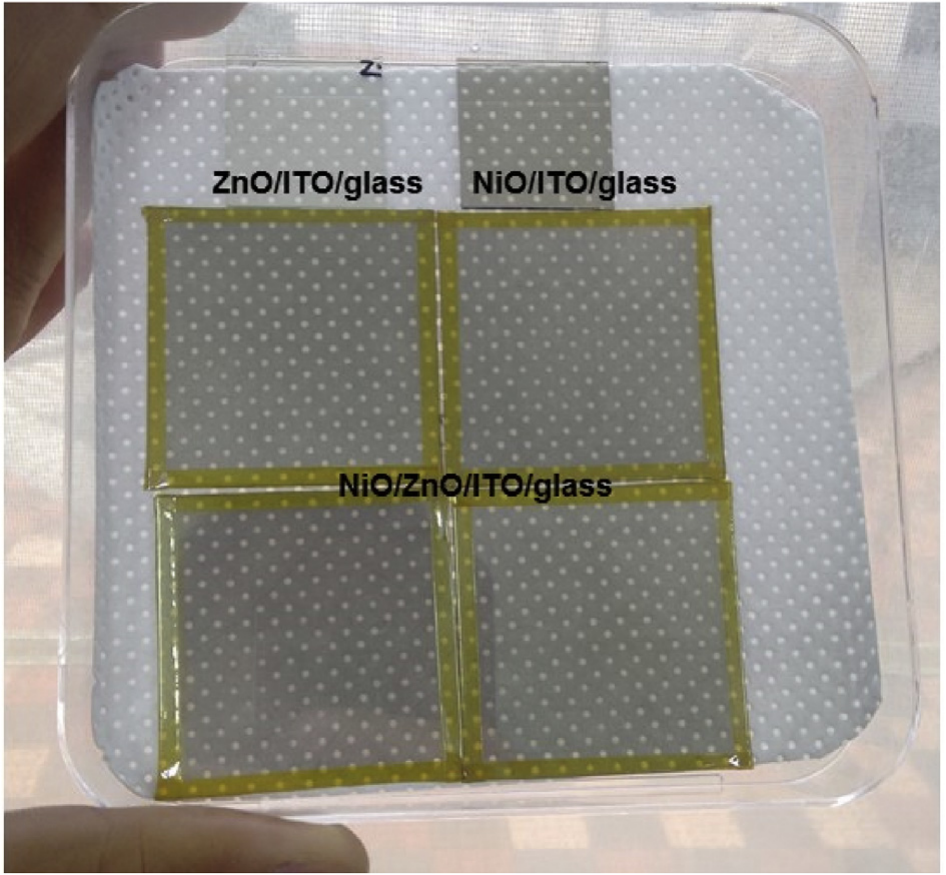
Large-area ZnO/NiO samples on ITO-coated glass. The yellow area is masked using Kapton tape. The reference samples of ZnO and NiO are prepared on the ITO/glass substrate.

**Fig. 2:**
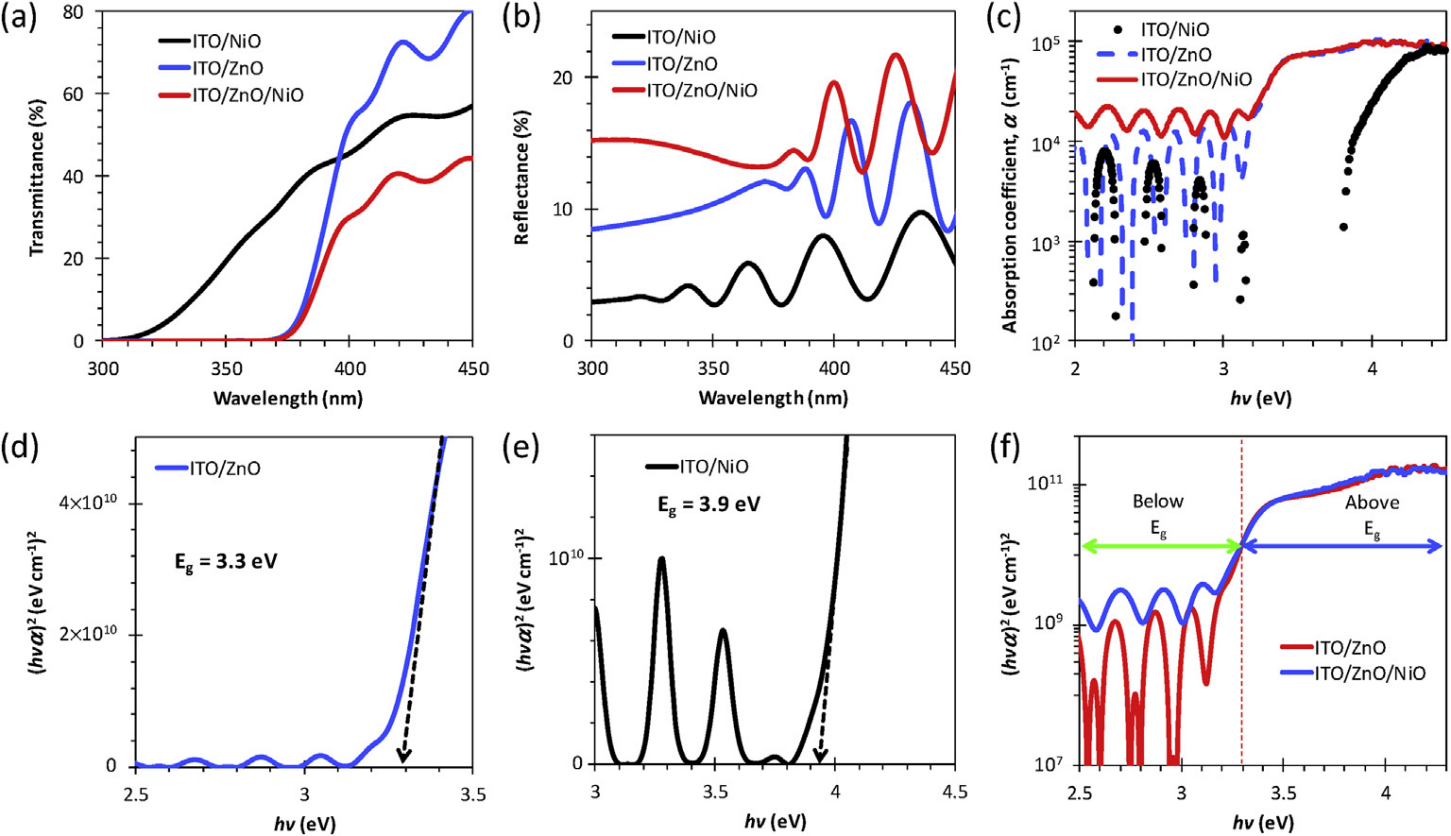
(**a**) Transmittance, (**b**) reflectance and (**c**) absorption coefficient of NiO, ZnO, and ZnO/NiO fabricated on ITO/glass. Tauc plot analysis: (**d**) ZnO, (**e**) NiO, and (**f**) ZnO/NiO samples on ITO/glass. Here, α values were estimated using the relation$\;\alpha \;\left(\text{λ}\right)=\;\frac{1}{t_{f}}\ln\left(\frac{{\left(1-R\left(\lambda \right)\right)}^{2}}{T\left(\lambda \right)}\right)$, where *t*_*f*_ is the film thickness.

**Fig. 3:**
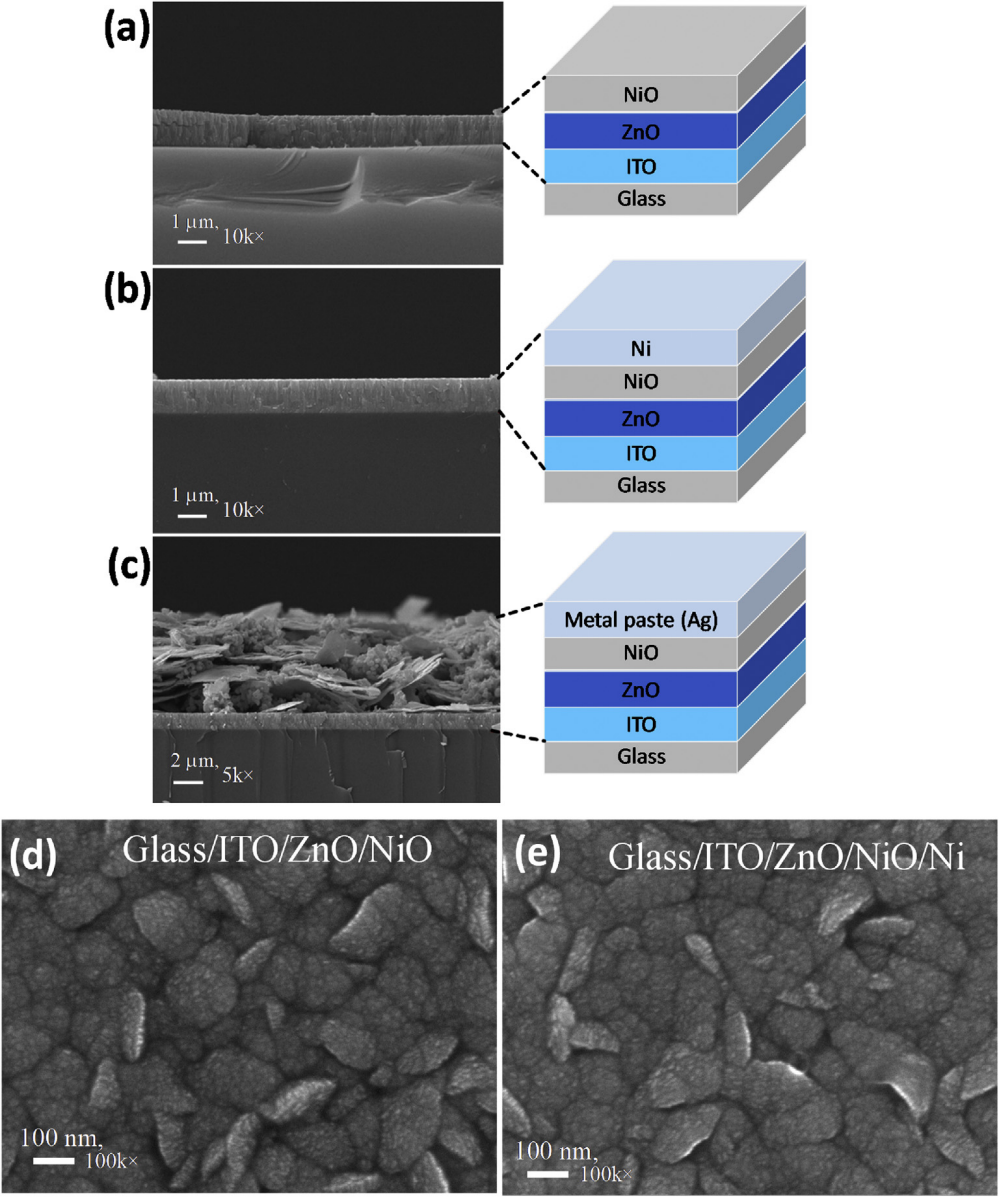
Cross-sectional images of (**a**) glass/ITO/ZnO/NiO, (**b**) glass/ITO/ZnO/NiO/Ni (sputtered), and (**c**) glass/ITO/ZnO/NiO/Ag paste. The device schematic is shown on the right of each FESEM image. Surface morphology of the device (**d**) before and (**e**) after Ni deposition using sputtering.

**Fig. 4:**
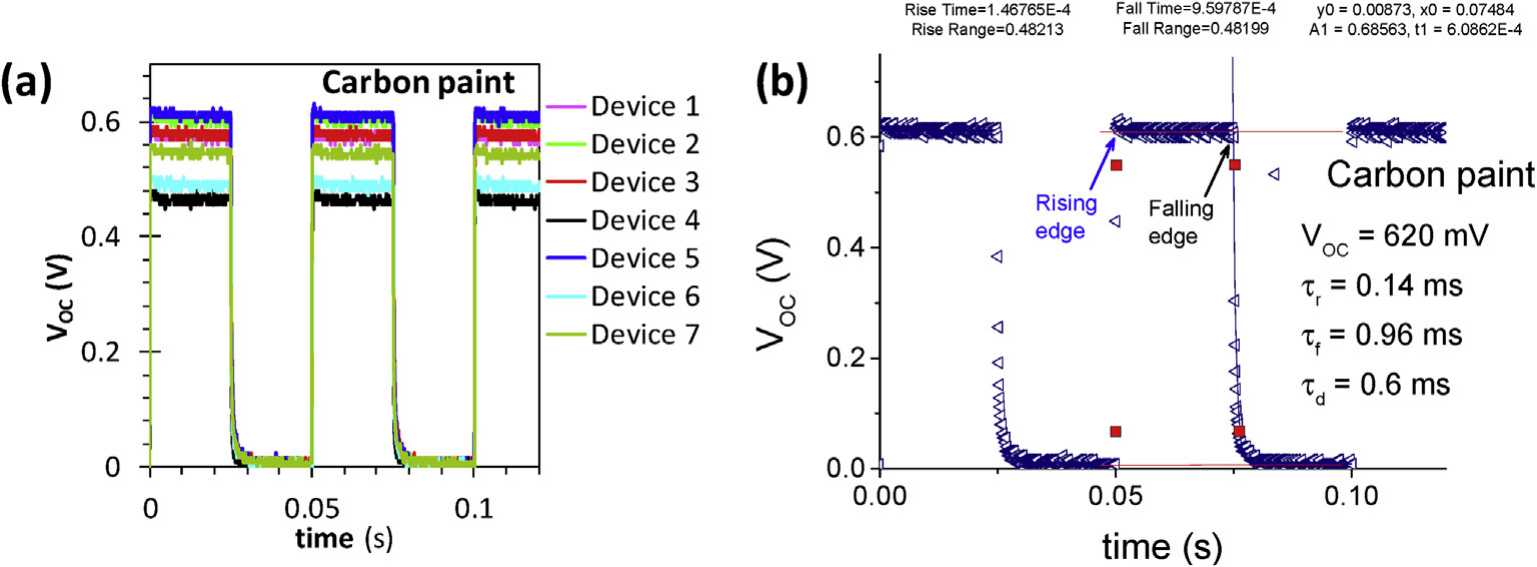
(**a**) OCVD analysis of the carbon paint/NiO/ZnO/ITO/glass device. A total of eight devices were analyzed. (**b**) Device with V_OC_ = 620 mV was analyzed in terms of rising time, falling time, and decay time. Here, the decay time was estimated using curve fitting of the exponential decay relation with the background offset, VOC(t)=yo+A1e−(x−xo)td, where y_o_, A_1_, and t_d_ are the background voltage, V_OC_ constant, and minority carrier lifetime, respectively. The fitting parameters y_o_, A_1_, x_o_, and t_d_ corresponding to the curve fitting function are noted in the inset.

**Fig. 5:**
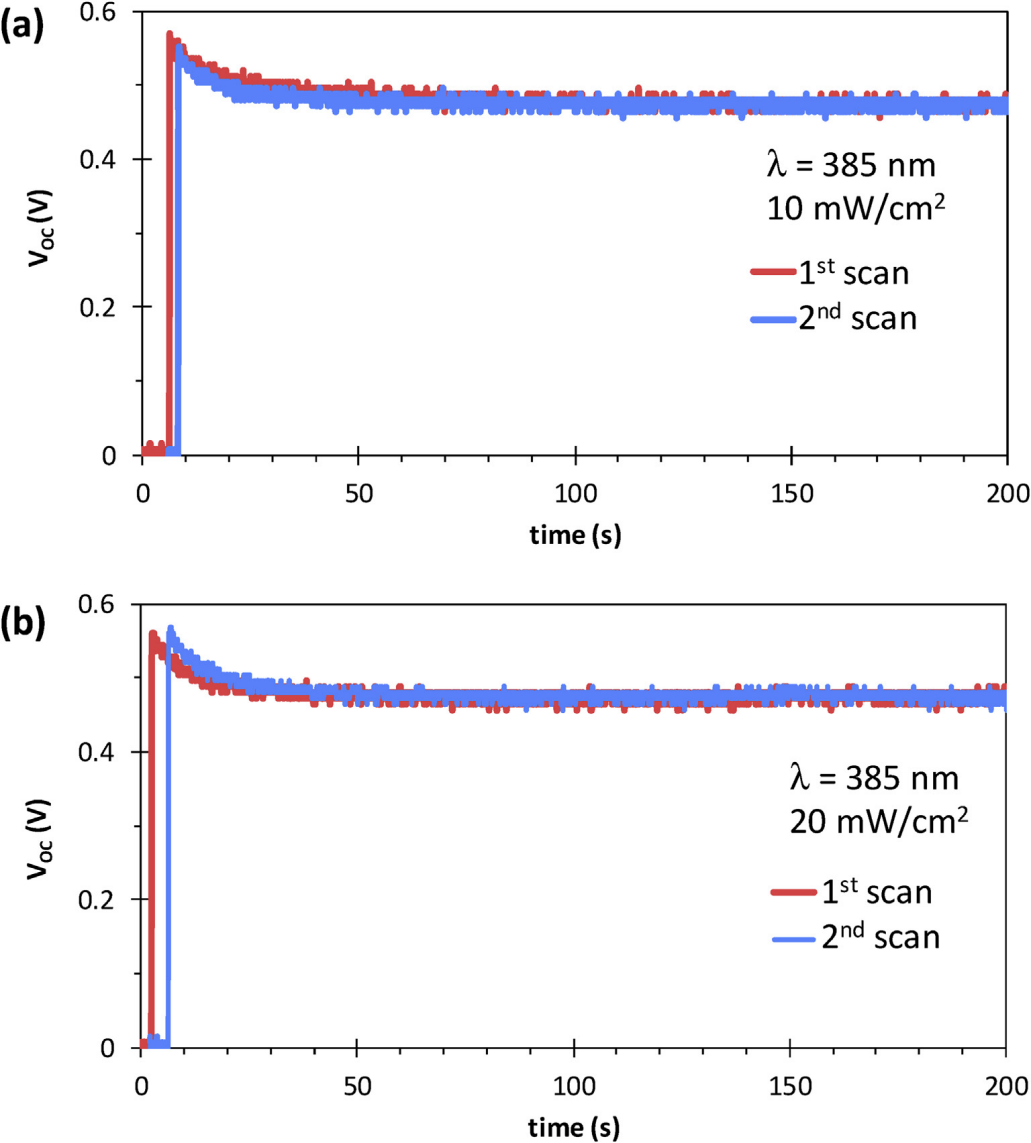
Prolonged V_OC_ of the glass/ITO/ZnO/NiO/Ag microink device. Stability, repeatability and effect of UV intensity (**a**) 10 mW/cm^2^ and (**b**) 20 mW/cm^2^ were examined.

**Fig. 6:**
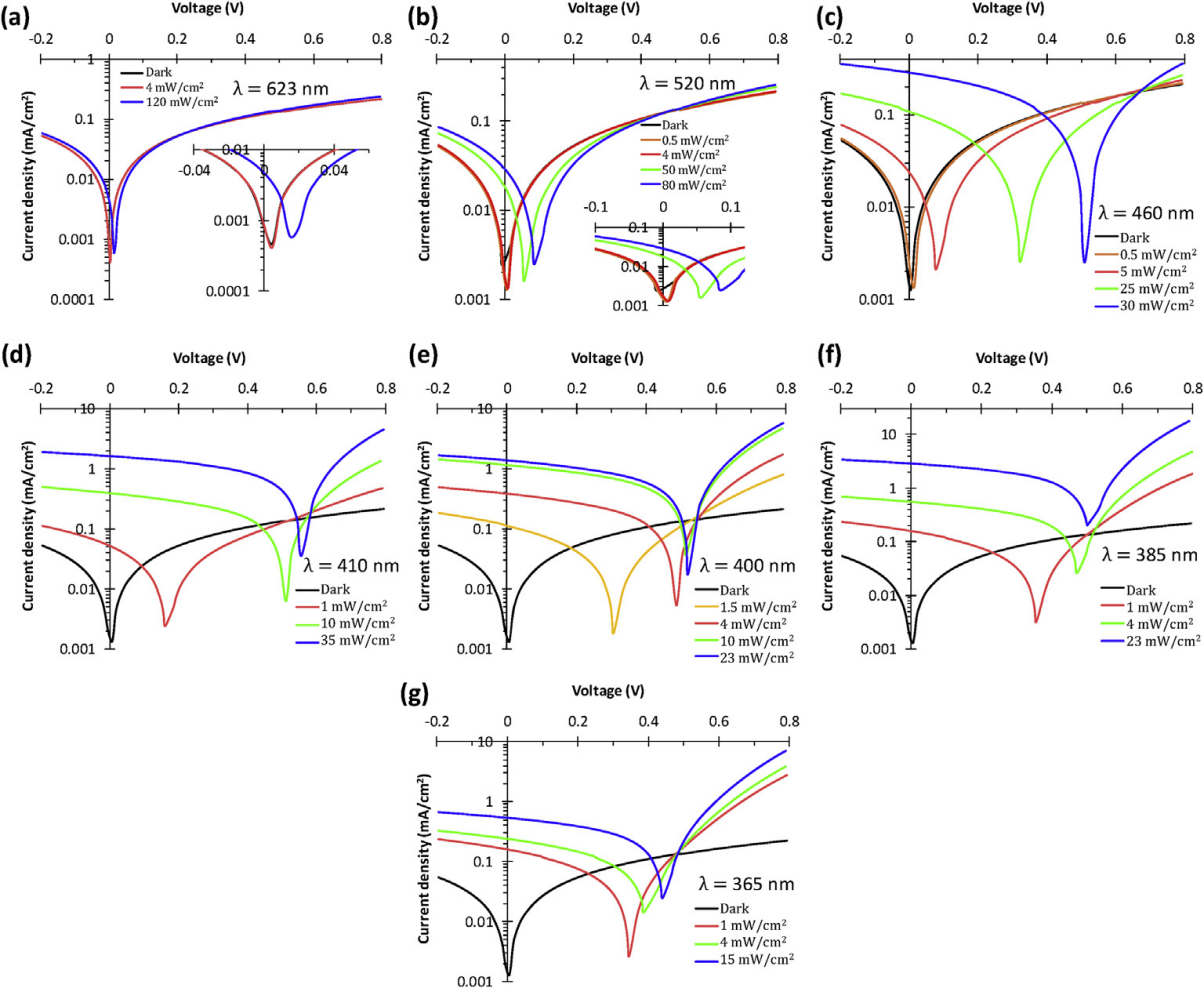
Current-voltage characteristics of the Ag microink/NiO/ZnO/ITO/glass device using light wavelengths of (**a**) λ = 623 nm (intermediate-band optical excitation) (inset: V_OC_ of 28 mV and zero-bias photocurrent), (**b**) λ = 520 nm (intermediate-band optical excitation) (inset: V_OC_ of 80 mV and zero-bias photocurrent), (**c**) λ = 460 nm (intermediate-band optical excitation), (**d**) λ = 410 nm (bound excitonic optical excitation), (**e**) λ = 400 nm (bound excitonic optical excitation), (**f**) λ = 385 nm (free-excitonic optical excitation), (**g**) λ = 365 nm (band-to-band optical excitation). Steady-state analysis (J-V characteristics) of the ITO/ZnO/NiO/Ag microink device (device area = 7.065 mm^2^).

Fig. 1 shows the large area samples of ZnO/NiO heterostructure grown at room temperature using by 4-inch sputtering [1]. The transmittance, reflectance, absorption coefficient and Tauc plot data of NiO, ZnO, and ZnO/NiO films on the ITO/glass are presented in Fig. 2. Further, the cross-sectional and surface morphology of the ZnO/NiO heterostructure is presented in Fig. 3 by using FESEM images. Estimated carrier lifetime of ZnO/NiO device from OCVD plots is shown in Fig. 4. Fig. 5 shows the solar cell performances. The light source of the wavelength of 385 nm was used to acquire these data. Fig. 6 shows the I–V characteristics plots of ZnO/NiO device under various wavelength of light illumination and its intensity dependent. Intermediated-band optical excitation induced I–V characteristics are shown in Fig. 6 a-c for the light wavelength of 623 nm, 520 nm, and 460 nm, respectively. Bound-excitonic optical transition induced I–V plots are shown in Fig. 6d and e for the light wavelength of 410 nm and 400 nm, respectively, while free-excitonic induced I–V plots are shown in Fig. 6f. Band-to-band optical excitation induced I–V plots are shown in Fig. 6g for the light wavelength of 365 nm.

## 2 Experimental design, materials, and methods

### 2.1 Sample preparation

The eagle glass was used as the substrate for the ITO/ZnO/NiO device fabrication and was cleaned prior to the fabrication process described in Ref. [1].

**Table:** The condition for ZnO layer deposition is presented as follow.

Target	ZnO (∅4 inch, purity 99.999%)
RF power	300 W
Gas/Flow rate	Ar, 50 sccm
Working pressure	5 mTorr
Temperature	Room temperature
Deposition time	60 minutes

**Table:** The condition for NiO layer deposition is presented as follow.

Target	Ni (∅4 inch, purity 99.999%)
DC power	50 W
Gas/Flow rate	Ar/O_2_, 30/4 sccm
Working pressure	3mTorr
Temperature	Room temperature
Deposition time	60 minutes.

**Table:** The condition for ITO layer deposition is presented as follow [2],

Target	ITO (∅4 inch, purity 99.999%)
RF power	300 W
Gas/Flow rate	Ar 50 sccm
Working pressure	5 mTorr
Temperature	Ambient temperature
Deposition time	15 minutes
Post-processing	Rapid thermal treatment, vacuum of 10^−2^ Torr, hold 550 °C for 15 minutes

### 2.2 Sample characterization

Samples of ZnO/NiO characterized in this data article are shown in Fig. 1. The transmittance, reflectance data of NiO, ZnO, and ZnO/NiO samples obtained using UV–visible diffused reflectance photo spectrometer are presented by Fig. 2a and b, respectively. Diffused integrating sphere was used to mount the samples. The absorption coefficient data of these samples are presented in Fig. 2c and their Tauc plots for the direct allowed optical transition are shown in Fig. 2d–f. The thickness of the device was measured from the cross-sectional images as shown in Fig. 3a–c, while the surface morphology of the device before and after Ni layer deposition is shown in Fig. 3d and e, respectively. These images were obtained by Field emission scanning electron microscope (FESEM, JOEL, JSM_7800 F). Carrier lifetime of the ZnO/NiO device was studied by OCVD analysis as shown in Fig. 4. These plots were obtained from the device with carbon paint under the pulsed light illumination of 385 nm and intensity of 7 mW/cm^2^. A function generator (MFG-3013A, MCH instruments) was applied to control the pulse rate and the light intensity. Stability, repeatability, and effect of UV light intensity of the fabricated ITO/ZnO/NiO/Ag microink device were studied by the prolonged V_OC_ characteristics as shown in Fig. 5. These data were obtained under the UV light wavelength of 385 nm with the intensity of 10–20 mW/cm^2^. I–V characteristics of the ZnO/NiO/Ag microink device under the various optical excitation in steady-state are shown in Fig. 6. The light wavelength of 623 nm, 520 nm, and 460 nm was used to obtain the intermediated-band optical excitation induced I–V plots as shown in Fig. 6a–c, respectively. Light wavelength of 410 nm and 400 nm was used to obtain the I–V plots from bound-excitonic optical excitation as shown in Fig. 6d and e, respectively. Further, the free-excitonic optical induced I–V plots obtained using a wavelength of 385 nm are shown in Fig. 6f. Finally, 365 nm wavelength of light was used to obtain the band-to-band optical excitation induced I–V plots as shown in Fig. 6g.

### 2.3 Carrier lifetime using open circuit voltage decay (OCVD)

The V_OC_ in a conventional solar cell is defined as the difference between the quasi-Fermi levels of electrons (E_fn_) and holes (E_fp_) at the respective selective contacts (ITO and NiO/Metal choice) (reference [3], [4]).

(1) $$\text{q}V_{oc}=E_{fn}-E_{fp}$$

where q, *E*_*fn*_ and *E*_*fp*_ are the elementary charge and the quasi-Fermi levels corresponding to the electrons and holes, respectively. This means that the V_OC_ value resides between the conduction band (E_c_) and the valence band (E_v_). The separation of quasi-Fermi levels corresponding to the photogenerated electrons (*n*) and holes (*p*) is fundamentally associated with the lifetime (τ) of these charges, which undergo a recombination process. This τ is a superposition of radiative and non-radiative recombination components, and can be determined by finding the small perturbation in $E_{fn}-E_{fp}$ after the light is switched off (transient state). Hence, τ can be estimated using V_OC_ decay and the following relation [3], [4].

(2) $${\text{τ}=\;-kT/q\;\left(\left(dV_{OC}\right)/dt\right)}^{-1}$$

where *k* is the Boltzmann constant (1.38 × 10^−23^ J K^−1^), and *T* is the absolute temperature. Moreover, V_OC_ as a function of E_g_ and charge-carrier density (electrons (*n*) and, holes (*p*)) can be written as the following.

(3) $$\text{q}V_{OC}=\;E_{g}-\;\text{ln}\left(N_{C}N_{V}/np\right)$$

where *N*_*C*_ and *N*_*V*_ are the effective densities of states of electrons and holes, respectively, which are constants. According to this relation, V_OC_ in the device depends on the number densities of photogenerated *n* and *p* and can reach as high as the value of *E*_*g*_ Refs. [3], [4].
